# Mobile Integrated Healthcare Intervention and Impact Analysis with a Medicare Advantage Population

**DOI:** 10.1089/pop.2017.0130

**Published:** 2018-09-26

**Authors:** Brooke Roeper, Jonathan Mocko, Lanty M. O'Connor, Jiaquan Zhou, Daniel Castillo, Eric H. Beck

**Affiliations:** Evolution Health, Dallas, Texas.

**Keywords:** population health, care management, cost of care, intervention, elderly

## Abstract

Mobile Integrated Healthcare (MIH) is a patient-centered, innovative delivery model offering on-demand, needs-based care and preventive services, delivered in the patient's home or mobile environment. An interprofessional MIH clinical team delivered a care coordination program for a Medicare Advantage Preferred Provider Organization that was risk assigned prior to intervention to target the highest risk members. Using claims and eligibility data, 6 months of pre-program experience and 6 months of program-influenced experience from the intervention cohort was compared to a propensity score–matched comparison cohort to measure impact. The intervention led to a reduction in inpatient and emergency department utilization, resulting in net savings amount totals of $2.4 million over the 6 months of the program. After accounting for the costs of implementing the program, the intervention produced a return on investment of 2.97. Additionally, high patient activation and experience lend strength to this MIH intervention as a promising model to reduce utilization and costs while keeping patient satisfaction high.

## Background

The US health care system has been increasingly impacted by high costs and variable quality, prompting reform through value-focused reimbursement models.^1^ Even with this shift toward value, health care expenditures continue to be higher in the United States than in other developed countries, while quality and health outcomes lag behind. One strategy for decreasing total health care costs is to reduce wasteful spending. The Institute of Medicine estimated that in 2009 the United States wasted $750 billion on unnecessary health spending, or roughly 30% of total health care costs for the country that year.^2^ There are many types of medical waste – overpriced drugs and procedures, medically unnecessary services, excessive administrative costs^3^ – and these complex issues are challenging to address. By understanding impactable areas of waste, health care stakeholders may begin to address this problem. As one example, in 2011 an estimated $25 to $45 billion was spent on ineffective transitions between settings or locations of care for patients.^4^ Managing chronically ill populations of high-risk elderly patients has been especially challenging because of this population's frailty; high emergency medical service (EMS), emergency department (ED), and inpatient services utilization; and high hospital readmission rates.^5^ An historic lack of post-acute transitional care services and the inability of patients to receive timely follow-up with their primary care physician outside of traditional business hours has led to a significant gap in the quality and access to care for this population.

Mobile Integrated Healthcare (MIH) offers an innovative approach to keep patients from falling through the cracks created by fragmented and disjointed care. Although there are numerous models and interventions demonstrating improved health outcomes and reduced costs,^6–10^ MIH offers a novel and emerging approach to coordinating care, reducing unnecessary medical spending, and improving quality.^11,12^ MIH is an alternate delivery model designed as a response to such gaps in care, serving patients in an outpatient setting by providing 24-hour/7-day needs-based, at-home care.^13–15^ More specifically, MIH offers patient-centered acute care, chronic care, and preventive services delivered in the home or mobile environment by synchronizing clinicians, infrastructure, and resources in a cost-effective manner.^13,16^ It is specifically designed to improve health outcomes, patient experience, and integration between systems of care while reducing health care costs for a defined population.

The value created by MIH interventions becomes apparent when examining how and where the US population currently accesses health care. Because of actual and perceived difficulties with scheduling access to primary care, particularly in off hours, patients are now seeking care elsewhere. More than a quarter of acute care visits now occur in hospital EDs, including almost all weekend and after-hours encounters.^17^ Studies suggest that approximately 15% of all Medicare beneficiaries transported to the ED by EMS were either nonemergent or emergent and primary care treatable, costing approximately $1 billion per year.^18^ Additionally, unplanned rehospitalizations cost Medicare $26 billion annually, with an estimated $17 billion spent on potentially avoidable readmissions.^19^ Emergency, urgent, or unplanned care also is often disconnected from the patient's ongoing health care management, resulting in additional financial burden related to duplicate testing, an increase in the risk of medical errors, and a lack of communication and coordination between care teams and settings.^17^ MIH interventions have the potential to close some of these gaps while decreasing cost and improving patient experience.

This study strengthens earlier findings that the MIH intervention model can demonstrate decreased ED and inpatient utilization and per-member per-month (PMPM) cost for a high-risk population while providing a positive experience for patients.^14^ Additionally, this retrospective study includes an interval cost-effectiveness evaluation. Specifically, this study analyzed an MIH care coordination intervention program at scale with a Medicare Advantage Preferred Provider Organization (MAPPO) population to quantify the cost savings generated, the reduction in avoidable utilization, and the associated impact on patient experience. The intervention's effectiveness also is evaluated by measuring its ability to drive positive change in patient activation (ie, an individual's ability to manage his or her own health and health care).^20,21^ This analysis used actuarial and epidemiological frameworks for the review of available paid claims, administrative, operational, and clinical data to assess the impact of the MIH intervention on health, experience, and cost.

## Methods

### Study setting

To be included in this retrospective observational study, participants needed to be an enrolled patient in the MIH care coordination program that recruited members from a MAPPO Florida-wide population under the care of FLOS, P.A. (a mobile in-home interprofessional medical practice, Florida Outpatient Services).^14^ There were 61,804 members in the MAPPO population at the start of the MIH intervention in November of 2015; 55% were female, the mean age of the population was 71.2 years, and the initial average Hierarchical Condition Category (HCC) risk score for the population was 1.07. The majority of patients resided in larger metropolitan cities such as Miami, Fort Lauderdale, and Tampa, though members were located across the entire state of Florida.

Internal and external proprietary predictive models identified and stratified high-risk members based on paid claims data. These members then were targeted by FLOS practice providers using several outreach methods: direct mail, telephone calls, and emails. Consenting members were enrolled in one of 3 evidence-based, interprofessional, clinician-delivered MIH intervention programs depending on whether they were (1) transitioning from one care setting to another (ie, transition of care [TOC] from hospital to home, hospital to nursing facility, nursing facility to home, and/or ED to home); (2) high-risk or chronically ill, needing longitudinal management (LHR); or (3) needing palliative support for advanced illness management (AIM) of chronic disease. Physician assistants or nurse practitioners, who were under physician supervision, created standardized care plans for all enrolled members that were used to guide team-based care coordination and intervention activities in collaboration with the member's primary care and/or specialist providers.

All consented and enrolled members had access to 24-hour/7-day/365-days per year services, accessible by telephonic hotline or text messaging, for on-demand unplanned care (UPC) needs. These unscheduled, unplanned member-initiated calls were triaged by nurses, using proprietary triage algorithms, and resulted in needs-matched, time-appropriate call navigation. UPC activity included: (1) an on-demand in-home clinician visit, (2) a telephonic consultation with a prescribing provider, social worker, or pharmacist, (3) a telemedicine encounter, and/or (4) a scheduled follow-up with an MIH clinician, in-network provider, or nonclinical support service (eg, transportation, advocacy, community resources). Both the MIH intervention programs (TOC, LHR, AIM) and the UPC services were delivered by the physician-led, interprofessional MIH team of emergency medical technicians, paramedics, nurses, social workers, pharmacists, and advanced practice providers.

Once enrolled, the high-risk members who were initially targeted using historical paid claims data and proprietary risk-prediction algorithms were then prospectively risk stratified using the Patient Activation Measure (PAM). This 13-item measure segments patients into one of 4 activation levels and is not condition-specific; it quantifies a person's ability to manage and advocate for his or her own health and requisite care based on his or her related knowledge, skills, and confidence.^20^ Significant evidence exists linking higher PAM scores to decreased costs and improved health outcomes and patient experience,^22^ and PAM has been shown to accurately predict health care costs and utilization.^23,24^ PAM also has evidence to support its effectiveness in guiding interventions for maximal return, as an intervention program process measure; as a leading indicator predictive of cost, utilization and experience; or as a population-level impact outcome.^23^ The members' PAM score and clinical intervention program (TOC, LHR, AIM) determined the scheduled amount of intervention activity (ie, weekly phone calls, in-home encounters, education) they received. The scope and content of planned scheduled intervention encounters was based on the member's diagnosis, clinical conditions, medication and care plan adherence, coordination needs, changes in member status, and PAM. Key intervention encounter themes included self-management coaching, outpatient appointment follow-up, medication instruction, diagnosis-related education, and reinforcement of available alternatives to 911, EMS, the ED, and the hospital that would allow a member to remain at home. In addition to this individualized care plan of scheduled encounters, members had 24-hour/7-day access to on-demand UPC services.

Consistent with a previously reported preliminary impact analysis, an engaged member is defined as any member who met all 4 of the following criteria: (1) the MIH care team spoke to (in person or telephonically) and (2) explained the clinical model, (3) offered enrollment, and (4) provided the UPC telephone number. An enrolled member is defined as any member who was engaged and subsequently consented to one of the MIH program interventions (LHR, AIM, TOC).^14^

### Study sample

This study builds on earlier experience and evaluation, with an additional 3 months of intervention and claim development enabling a more accurate assessment of program impact as well as interval cost-effectiveness results. The principle data sources for this study were the enrollment and medical claims files for the statewide MAPPO membership from May 2015 through November 2016. This time period allowed the identification and sufficient measurement of the membership's PMPM incurred and paid claim costs as well as the corresponding utilization trend for a 6-month preintervention and 6-month postintervention period, with 4 months of run-out held constant for claims development. Intervention care plans were designed to reduce potentially avoidable medical spending such as unnecessary ED visits and inpatient medical admissions. As such, specific “cost categories” were used to group claims and properly allocate impact. The cost categories were defined by Diagnosis-Related Groups, revenue codes, Current Procedural Terminology, 4th Edition codes, and provider-type codes.

Outreach, engagement, and enrollment information was tracked using the FLOS practice's internal logistics operating and scheduling platform. Each clinical engagement, outreach, and intervention encounter was logged and tracked using this platform, enabling accurate monitoring of all intervention activities, time on task, and real-time cost accounting of those activities.

For initial and ongoing member risk prediction and outreach targeting, proprietary internal tools as well as Milliman PRM Analytics^25^ software were used to identify those members predicted to be at risk for high impactable costs, defined as costs that are potentially avoidable. Two years of pre-program historical paid claims data for the entire population were analyzed to establish a baseline for the population. Members' baseline characteristics, utilization, and PMPM costs were collected from the health plan's enrollment data and claims. The claims were filtered to include only ED and inpatient medical spend. These members were then placed into separate risk cohorts (levels 1, 2, and 3) by predicted utilization and impactable cost calculation – with level 1 being those members with the highest impactable costs as predicted over the next 6 months. The MIH program specifically targeted those in risk levels 1 and 2. Outreach calls were then placed to those targeted members with the goal to enroll them into one of the MIH intervention programs.

### Analysis of data

Members enrolled in the MIH intervention from November 2015 through February 2016 were assigned to the intervention cohort. A control group was designated to estimate medical trend for like-matched members within the MA population who did not participate in the intervention. To identify and select the control cohort, the control reference pool was restricted to members who were initially targeted for intervention but either declined services or were never reached. These were level 1 and level 2 members from the initial claims-based stratification of the population wherein members were ranked based on predicted hospital and ED utilization, and potentially impactable medical spend estimates, within the subsequent 6 months. Propensity score matching was then used to isolate the control group to like matches within the intervention cohort. The following dimensions were used within the scoring to align the control and intervention cohorts: HCC risk score, age, probability of an ED visit, probability of an inpatient admission, sex, number of chronic conditions, congestive heart failure, diabetes, dementia, chronic kidney disease, coronary artery disease, and cerebrovascular accident/transient ischemic attack. The propensity score matching was calculated using the R statistical software (R Foundation for Statistical Computing, Vienna, Austria); R is an open-sourced free software environment and programing language for statistical computing and graphics.^26^ Logistic regression was used to estimate the propensity scores for each member within the control reference pool and the intervention cohort. Aligned members from each set were matched and ranked according to similarity, achieving a statistically equivalent reference sample.

Statistical hypotheses testing on the 2 cohorts, control and intervention, was run to test for difference between the cohorts. Tests were run on all the data dimensions used to drive the propensity scores, and there was no statistically significant difference between the 2 cohorts (Table 1). Members enrolled in the intervention cohort were those who consented to the program and received a scheduled welcome visit. The start date was defined as the date of their welcome visit – the first in-person interaction with an MIH clinician. The control cohort was established from those members who were targeted for engagement but did not complete a welcome visit, and thus were never categorized as enrolled. These members were assigned a start date of January 1, 2016, as this provided 6 months of pre-program data to be analyzed as well as 6 months of program data. Using this date also minimized potential impact from seasonality, as the mean welcome visit of the enrolled members was January 1, 2016.

**Table 1.** Baseline Characteristics of Intervention and Control Cohorts

	*Intervention (*n* = 1074)*	*Control (*n* = 1241)*	P *value*
Demographics			
Age, mean	73.56	74.63	0.0700
Females	58.4%	57.0%	0.5400
HCC Risk Score, mean	3.03	3.03	0.4362
PAC (6 months)	$6633	$6342	0.4567
Health Status			
Congestive Heart Failure	24.0%	23.0%	0.7300
Chronic Obstructive Pulmonary Disease	38.0%	39.0%	0.6540
Diabetes	47.0%	46.0%	0.6565
Dementia	13.0%	14.0%	0.3315
Chronic Kidney Disease	31.0%	31.9%	0.7764
Coronary Artery Disease	43.0%	46.0%	0.1861
Cerebrovascular Accident/Transient Ischemic Attack	20.3%	22.9%	0.1602
Chronic conditions, mean	12.8	12.89	0.7353

HCC, Hierarchical Condition Category; PAC, potentially avoidable cost.

A pre–post risk score-adjusted analysis was completed that accounted for the intervention cohort's utilization and PMPM paid claims trends from the 6-month baseline period before program implementation through the 6-month intervention period. For each 30-day interval of incurred claims, run-out was held to 4 months to maintain a consistent completion level between periods. This was compared against the control cohort's experience over the same time period, using the same completion and calculation methodology. The claims data for each cohort consisted of total medical spend (facility and professional) for all ED services and all inpatient medical admissions (facility and professional), along with emergency transport spending. Program savings were estimated using risk-adjusted trend over the 360-day experience period for the control cohort, and applying that trend to the intervention cohort to derive an estimated spend without intervention. Matching encounter information to estimated savings per 30-day increment, an estimated savings per encounter type was calculated.

A set of 1-tailed paired Student *t* tests was used for PMPM calculations, utilization outcomes, and readmissions comparisons. For the readmission subanalysis, the trend differences between the 2 cohorts were examined and 1-tailed paired Student *t* tests were performed.

## Results

### Descriptive characteristics

The demographic characteristics of both the intervention (N = 1074) and control (N = 1241) cohorts are presented in Table 1. Given that the control cohort was propensity score matched to the intervention cohort for the purposes of this study (HCC risk scores, demographics, and potentially avoidable costs), the study team finds there to be no significant difference between the 2 cohorts in terms of sex, age, risk score, or comorbidities, and therefore, the groups are appropriate for comparison.

### Costs, utilization, and cost-effectiveness analysis

The PMPM costs and utilization per 1000 of the intervention and control cohorts are presented in Table 2. These utilization differences correlated to the observed differences in PMPM costs between the intervention cohort and matched control, and represent estimated savings per patient. Table 3 demonstrates the savings estimated using the actuarial-adjusted historical control methodology. The study team started with the risk-adjusted PMPM for the intervention cohort over the 6-month preintervention period ($359.59) and projected that forward using the risk-adjusted trend from the control cohort (4.1% per month). The team then applied the risk score back to the risk-adjusted trended PMPM for the intervention cohort and deducted the actual PMPM for the group to calculate savings. This net savings amount totals over $2.4 million over the 6 months of the program.

**Table 2.** Per-Member Per-Month Costs, Utilization, and Trends: Risk Adjusted Per-Member Per-Month and Utilization per 1000

	*pre*	*post*	*diff*	*pre*	*post*	*diff*	
	*Intervention (*n *= 992)*			*Control (*n *= 995)*			P *value*
PMPM (6 mo. mean)							
Total	$359.59	$317.77	$−41.82	$228.81	$291.98	$63.17	0.00002
Inpatient	$179.65	$162.83	$−16.82	$100.66	$152.45	$51.78	0.01538
ED	$28.80	$23.44	$−5.36	$14.50	$18.13	$3.63	0.00687
Utilization (6 mo. mean)							
Inpatient (per 1000)	28.14	22.23	−5.91	15.63	21.40	5.77	0.00001
ED (per 1000)	39.47	30.19	−9.28	19.64	24.26	4.62	0.00280

ED, emergency department; PMPM, per member per month.

**Table 3.** Estimated Program Savings and Cost–Benefit Analysis

		*Post-Program Engagement*						
		+*30 days*	+*60 days*	+*90 days*	+*120 days*	+*150 days*	+*180 days*	
Estimated Savings related to reduced PMPM								
Intervent. Avg Post Risk-Adj PMPM X 6 mo Control Post Cost Trend Risk Adj(4.1%)		$359.59 × 1.041^3.5^ = $413.89	$430.86	$448.52	$466.91	$486.06	$505.99	
Risk Score		3.03	2.99	2.98	2.94	2.88	3.07	
Unadjusted PMPM		$1252.88	$1288.18	$1336.96	$1375.05	$1401.45	$1551.51	
Minus:	Actual Int. Cost PMPM	$1108.19	$977.29	$821.92	$903.00	$928.02	$949.42	
Equals:	Estimated Savings PMPM	$144.69	$311.65	$515.03	$472.05	$473.43	$602.09	
Multiplied by:	Membership	1056	1017	978	935	955	898	TOTAL SAVINGS:
Monthly Estimated Savings		$152,792	$316,948	$503,704	$441,371	$452,121	$540,676	$2,407,612
		−35%	−24%	−39%	−34%	−34%	−39%	
TOTAL COSTS of IMPLEMENTATION: (salary + benefits of providers, mileage costs and% of fixed costs)								$810,000
Return on Investment:								2.97

Adj, adjustment; Avg, average; Intervent, intervention; PMPM, per member per month

Table 3 displays the cost–benefit analysis of the program. The aforementioned savings are compared with the initial cost to outreach, engage, and enroll members as well as monthly participation costs. These data were collected from intervention activity task time documented in the internal logistics operating and scheduling platform, and represent the costs calculated from salary and benefits of the providers, average drive time allocation for mileage costs, and appropriate allocation of fixed overhead costs. Adding these together over the 6-month intervention period resulted in $810,000 of implementation costs. Using the data from Table 3, the study team determined that the implementation of this MIH intervention program for this high-risk MAPPO patient population yielded a return on investment (ROI) of 2.97.

### Readmissions

Although this study focuses on overall costs and utilization, subanalysis examined the readmission rate of the intervention and control cohorts. Readmission data were determined using paid claims data and are defined for the purposes of this study as an unadjusted 30-day hospital readmission rate for members who were hospitalized at an acute care hospital and had a subsequent unplanned readmission for any cause to an acute care hospital within 30 days of discharge. Although both cohorts were propensity score matched prior to the study, the intervention cohort did have higher readmissions per 1000 members pre intervention. Over the course of the program, the 30-day readmission trend was increasing at 14% for the control cohort and decreasing by 2.7% for the intervention cohort. This analysis is consistent with the aforementioned overall ED and inpatient medical cost and utilization reduction, as a lower number of readmissions also would contribute to lower ED and inpatient medical cost and utilization.

### Patient activation and experience

Patient activation scores were collected for the population at initial encounter and also serially during the course of the intervention, either at every 60 days or when there was a change in the patient's condition, such as an admission or increase in risk score. The PAM instrument provides a granular 0–100–point score based on patient responses, which is then utilized to assign the patient to one of 4 activation groups, with those in group 4 being the most activated. For the purposes of this analysis and consistent with PAM tool recommended calculation methodology, all initial PAM level 4s were removed from the PAM analysis as it is the increase in PAM that was of interest. Table 4 shows that for this high-risk intervention cohort, PAM scores increased 7.5%. This supports the aforementioned savings findings shown, as numerous publications have demonstrated that savings can be attained by improving the patient's engagement and activation.

**Table 4.** Intervention Cohort Change in Patient Activation Measures

Mean initial PAM Score	56.99	
Latest PAM Score	61.24	
Change	4.25	
% Change	7.5%	*P* < 0.01

377 patients with 2 or more PAM scores assessed.

PAM, Patient Activation Measure.

Although lowering costs and utilization are important goals of any value-focused care coordination and disease management program, member experience also remains a vital aspect of overall program impact and sustainability. Member satisfaction was high, as anonymously measured by a third-party compilation and analysis of member surveys, at the initial encounter and serially every 60 days while the member was enrolled in the MIH intervention.^27^ As noted in Table 5, 96% of members agreed or strongly agreed that providers communicated clearly and more than 86% would recommend their provider and the program to their family and friends.

**Table 5.** Intervention Cohort Patient Experience Results

	*Agree*	*Strongly Agree*	*Total*	*Statements*
Communication	21.88%	74.27%	96.15%	My provider actively sought my opinion; my provider listened carefully; my provider clearly communicated my options
Knowledge	16.75%	80.46%	97.21%	My provider was knowledgeable
Overall Satisfaction	9.91%	76.42%	86.33%	I would recommend my provider to family and friends

615 Surveys between Feb 15 and Oct 31.

### Causality

**FIG. 1.**
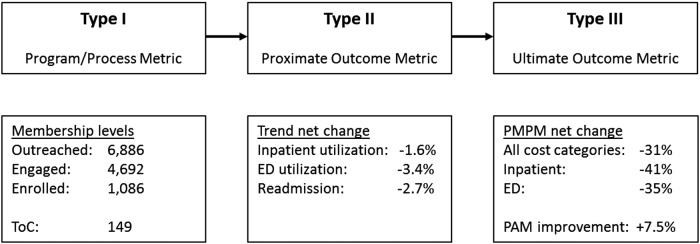
Causal pathway metric types and corresponding data. ED, emergency department; PMPM, per member per month; ToC, transitions of care.

Typically, most population health and care management interventions are not evaluated based on double-blind randomized controlled trials. Given that the primary outcome of interest was financial savings, something that did not occur, the study team utilized established methods from epidemiology, health services, observational, and outcomes research to ensure measurement validity. Wilson and MacDowell's^28^ “causal pathway” nomenclature and defined Type I, Type II, and Type III metrics for evaluating intervention causality in health program analysis was used to asses this MIH intervention's influence on PMPM savings. Figure 1 outlines this causality in terms of Type I, Type II and Type III metrics. For the Type I (Program/Process) metric, the study team includes the activity to target, enroll, and care for members in this MIH intervention. For the Type II (Proximate Outcome) metric, the team examined both the utilization reduction to ED and inpatient medical cost categories, as well as the readmission trend and PAM score improvement. These Type II metrics align with the ultimate outcome metric (Type III) of PMPM savings and support a likely causal relationship between the MIH intervention and the PMPM savings described.

## Discussion

This impact analysis of an MIH program for a MAPPO population supports the notion that such intervention programs can improve outcomes, improve the patient experience, and reduce costs at the population level. Previous reports on this intervention program were limited regarding quantification of required intervention resource and infrastructure cost. Resource and capacity utilization, start-up costs, and task time analysis are critically important to accurately reflect operating expenses associated with MIH intervention delivery. Specifically, the TOC, LHR, and AIM programmatic components, resource and infrastructure costs, as well as analysis of the ROI for the intervention are important additional findings shared in this quantitative analysis.

This MIH program evaluation compared the population's health care utilization based on historical actuarial analysis as well as epidemiologic methods to contemporaneously compare the intervention and control cohorts. Meaningful decreases in cost and utilization reported here are consistent with previously published experience quantifying impact of similar component interventions delivered as part of this MIH program. Specifically, the evidence-based interventions core to this MIH program included timely transitional care support with comprehensive medication review, scheduled self-management coaching and education for those at highest risk, timely referral to palliative care, and 24-hour/7-day on-demand access for acute and UPC needs.

The intervention outlined focused on a subpopulation of members at the highest risk for worse outcomes and high costs. Recently, the Bipartisan Policy Center projected 3.65 million Medicare beneficiaries meet the criteria of having 3 or more chronic conditions with functional or cognitive impairment. Further, it was estimated that these beneficiaries incur approximately $30,000 of Medicare costs per year.^29^ Much of this cost has been categorized as wasteful or potentially avoidable with improved care coordination and disease management. In order to control health care costs while improving quality, there will need to be further innovative models that focus on this high-need population.

The epidemiologic framework used in this analysis to consider causal association between this MIH intervention and the demonstrated changes in utilization and cost is maintained from an earlier publication. Key dimensions in considering potential causal inference in an observation setting include: strength of association, temporality, consistency, theoretical plausibility, specificity, dose–response relationship, experimental evidence, coherence, and analogy.^30^

The interpretation of the data and results presented here are consistent with the causal inference framework outlined. Previous literature has established the evidence base underpinning the content of the MIH intervention component activities; specifically, TOC support, PAM coaching for activation, advanced illness and high-risk member care coordination. Given the statistical significance of this interval analysis of utilization and cost, and that the notable change in trend is isolated to the intervention cohort following the institution of the intervention program, the study team finds good support for causal association. More specifically, there is strength in the association, temporally the intervention precedes the impact, and intervention outcomes are theoretically plausible based on previously reported evidence. Of note, consistent with the underlying intervention activities, the isolated and notable impact in the intervention cohort is unambiguous.

Improving health care experiences, access, and outcomes while containing per capita costs will continue to be an important focus for the Centers for Medicare & Medicaid Services, payers, at-risk providers, and other financiers of health services. This is especially notable considering the recent announcement of the National Health Expenditure Projections 2016–2025,^31^ projecting an average annual rate growth of 5.6% over that term and representing 19.9% of GDP by 2025 – a number that most economists believe is not sustainable. The continued importance of quantifying the value of innovative delivery models that demonstrate lowering costs while improving overall quality will be relevant to health policy discussions. This paper augments a previous analysis of the impact of a statewide MIH program on an MAPPO population.^14^ It furthers previous evaluation demonstrating improved outcomes and lower costs with more robust coordination of care, chronic disease management, and improved TOC. The results presented demonstrate that the interprofessional MIH intervention was strongly associated with statistically significant reductions in PMPM costs and utilization per 1000 members for ED and inpatient medical categories for the enrolled intervention cohort when compared to a like-matched control. This impact analysis, inclusive of cost-effectiveness, is the most robust and comprehensive evaluation of the MIH model's potential for cost reduction and outcomes improvement to date.

### Limitations

As noted with the preliminary impact analysis, selection bias may have been introduced because members self-selected to enroll in the program. However, both the intervention and control cohorts were identified using the same targeting algorithms and potentially avoidable cost values, as well as the other dimensions of comparison including HCC, age, sex, and chronic conditions – all of which were similar (Table 1). It is also worth noting that refusal to participate can be a marker for nonadherent patient behaviors, neither of which is well defined by a PAM score.

Regression to the mean also has been considered, given the high risk of the members targeted for intervention. Of note, regression to the mean is mostly an individual phenomenon rather than a population-level observation. Any regression to the mean should be approximately equal in both groups given that the reference population used as a control in this study was derived using the same objective criteria and that equivalence has been demonstrated between the cohorts.

These results are still developing as this program evaluation represents 6 months of intervention experience with 4 months of claims run-out. The full impact of the intervention will continue to be realized as the program matures. Differences between the actuarially expected projections and the actual incurred and paid amounts for all groups studied depend on the extent to which future experience conforms to the assumptions made for this analysis. It is certain that actual experience will not conform exactly to the assumptions used in this analysis.

In performing this analysis, the study team relied on data and information provided by the MAPPO payer and FLOS provider practice, consistent with industry standards and regulatory requirements. Also, as part of the analysis, the team relied on the payor to perform claims casing and adjudication. The team performed a limited review of the data integrity used directly in this analysis for reasonableness and consistency and have found no material defects. Of note, exact claim liabilities will only be determinable after a significant passage of time.

## Conclusions

Reining in health care spending for medically costly and complex individuals continues to be a primary value level in policy and reform discussions. Innovative delivery models such as MIH offer much promise as they may lower inpatient medical and ED utilization and costs, as well as lower readmissions. This analysis offers additional support in establishing the value of patient activation-specific targeting and intervention, the scalability of interprofessional team-based care, and the effectiveness of the MIH model in reducing avoidable costs and utilization in high-risk populations.
